# Pupil dilation predicts individual self-regulation success across domains

**DOI:** 10.1038/s41598-021-93121-y

**Published:** 2021-07-12

**Authors:** Silvia U. Maier, Marcus Grueschow

**Affiliations:** 1 Zurich Center for Neuroeconomics, Department of Economics, University of Zurich, Bluemlisalpstrasse 10, 8006 Zurich, Switzerland; 2 Neuroscience Center Zurich, University of Zurich, Swiss Federal Institute of Technology Zurich, Zurich, Switzerland; 3 Translational Neuromodeling Unit, Institute for Biomedical Engineering, University of Zurich and ETH Zurich, Zurich, Switzerland

**Keywords:** Psychology, Human behaviour, Neuroscience, Emotion

## Abstract

Multiple theories have proposed that increasing central arousal through the brain’s locus coeruleus—norepinephrine system may facilitate cognitive control and memory. However, the role of the arousal system in emotion regulation is less well understood. Pupil diameter is a proxy to infer upon the central arousal state. We employed an emotion regulation paradigm with a combination of design features that allowed us to dissociate regulation from emotional arousal in the pupil diameter time course of 34 healthy adults. Pupil diameter increase during regulation predicted individual differences in emotion regulation success beyond task difficulty. Moreover, the extent of this individual regulatory arousal boost predicted performance in another self-control task, dietary health challenges. Participants who harnessed more regulation-associated arousal during emotion regulation were also more successful in choosing healthier foods. These results suggest that a common arousal-based facilitation mechanism may support an individual’s self-control across domains.

## Introduction

Self-control skills enable individuals to align their behaviour with own long-term goals and values. Being able to experience different emotional states and if necessary, adaptively regulate emotional reactions, are core aspects of daily human lives^1,2^. Disturbances of emotion regulation are a hallmark of multiple psychiatric disorders^3–5^ and emotion control has recently been identified as the component of self-control that is most predictive of mental health^6^. For example, individuals who engage more automatically in reappraisal when encountering negative experiences may mitigate stress-inducing effects^7^ through this emotional buffer^8^. Despite the abundance of psychological disorders involving maladaptive emotion regulation^9^, validated quantitative tools to assess an individuals’ engagement in regulation in a clinical or research settings are surprisingly scarce and urgently needed^10^.

Novel frameworks conceptualize how emotion regulation operates on a moment-to-moment basis^11,12^ and across biological and psychological levels. However, it is not trivial to measure whether individuals engage in regulating their emotions at any given moment, and most importantly, to predict how successful they will be. These questions are paramount for both basic and applied research, because inflexibility or inability to adaptively regulate emotions through strategies that favour beneficial behaviour in the long term is a hallmark of diseases such as depression, eating disorders, substance abuse, and posttraumatic stress disorder for review see^13^, and similar impairments of cognitive and emotion control have also been reported and recently been linked to pupil dilation in ADHD^14–17^ and autism^18^.

We therefore aimed to simultaneously quantify the onset and efficacy of human emotion regulation. To this end, we combined an established emotion regulation paradigm^19–26^ with pupillometry—a measure intricately linked to activity in the arousal system^27–29^. Its millisecond temporal resolution allowed us to assess at which time point individuals engaged in emotion regulation. One crucial problem for the interpretation of such physiological readouts in emotion regulation research is whether an increase in pupil dilation relates to emotional arousal induced by the stimulus content or to engagement in genuine cognitive control processes in service of regulation. To dissociate these two aspects, we employed a combination of design features in the emotion regulation paradigm that allowed us to separate regulation-associated from emotional arousal components in the pupil diameter time course. Moreover, we aimed to predict from individual pupil diameter during the regulation period to which degree participants managed to render their emotions more neutral. Our findings present a physiological account based on pupil dilation that quantifies an individual’s engagement and success in emotion regulation. Furthermore, we additionally test whether this measure captures features of an individual’s regulation ability that generalize to another self-control domain^30^, namely dietary health challenges.

That the pupil dilates in response to emotionally relevant stimuli has been corroborated by ample evidence^31–44^. Individual differences in pupil dilation in response to emotional stimuli show excellent test–retest reliability^36,45^. Pupillometry allows quantifying the pupil dilation response precisely, and pupil dilation and constriction have been related to activity in the sympathetic and parasympathetic branches of the autonomic nervous system^46–49^. Lesion studies show that the parasympathetic pathway primarily controls the pupil light reflex, i.e. pupil constriction in response to brightness changes, whereas the sympathetic pathway controls dilation in response to arousal^46^. Arousal could for example be caused by emotionally salient stimuli, but also be recruited in order to meet cognitive demands^50–56^ or in order to mobilize effort^27,57,58^. Previous studies have also reported that pupil diameter increases when individuals regulate their emotions^21,59–64^ or anticipate having to do so^34^. In addition, recent findings offered new insights into the basic mechanisms of emotion processing and cognitive control, by demonstrating that exerting executive control modulates both the parasympathetic and sympathetic branch of the autonomic nervous system to increase sympathetic activity and attenuates the parasympathetic pupil light reflex as well as the emotional arousal reaction measured in the pupil in response to negative stimuli^35^. These results showcase the importance of noradrenergic arousal as a proxy for and potential facilitator of cognitive control^51,52^.

During mental problem solving, pupil dilation is strongly correlated with the difficulty of the problem, as Hess and Polt^65^ first described. They suggested a measure of “total mental activity”, reflected in a combination of the latency and the amplitude of the pupil response, that we mimic in our approach. Kahneman and Beatty^66^ elegantly demonstrated how the pupil dilates in response to processing load and constricts once the load is reduced (see Beatty^67^ for review of earlier works and van der Wel and van Steenbergen^68^ for a recent review on pupil dilation during cognitive control).

Therefore, we assume that the pupil dilation signature during emotion regulation is informative about the individual cognitive engagement during this self-control process. We chose to investigate the engagement in regulation via pupil dilation because the ability to engage self-control is fundamental for how well individuals can follow through on their goals, which is hard to assess in an unbiased fashion via self-report. Because the participant cannot covertly manipulate pupil dilation, it provides an unbiased readout of the cognitive engagement in self-control, whereas self-reports have been shown to suffer from several subjective biases^69,70^. For elementary cognitive control processes such as updating, attention shifting, action inhibition and explore-exploit trade-offs, an adaptive modulation via arousal has been reported and measured using pupil dilation (see van der Wel and van Steenbergen^68^ for review). We thus hypothesized that individuals who increase their cognitive engagement and hence arousal levels to best meet the task requirements should be more successful regulators. We set out to test whether individuals who are able to boost task-relevant, adaptive processing through arousal (quantified here using pupil dilation) achieve higher levels of emotion control^51^. In our experiment, we demonstrate that this relationship holds for emotion regulation, and critically also transfers to another complex behavioural task, namely solving dietary health challenges. These two tasks employ important mechanisms associated with a wide array of disorders of affect and interpersonal conduct, obesity, and addiction^71,72^.

Using the pupil dilation signal, we predict how successful individuals apply an instructed regulation strategy, reappraisal. To provide a generalizable account, we investigate commonalities in the process of regulating emotional experiences of positive and negative valences. We show that the Pupil Dilation Index predicts individual differences in regulation success. Moreover, we demonstrate its convergent validity, as the Pupil Dilation Index measured in the emotion regulation task is associated with individual regulation success in a separate dietary health challenge. This strongly suggests that the Pupil Dilation Index captures a more general process supporting adaptive behaviour and regulation success across multiple task domains and may serve as a promising measure in future investigations of self-control mechanisms in health and disease.

## Results

### Emotion reappraisal task

**Figure 1:**
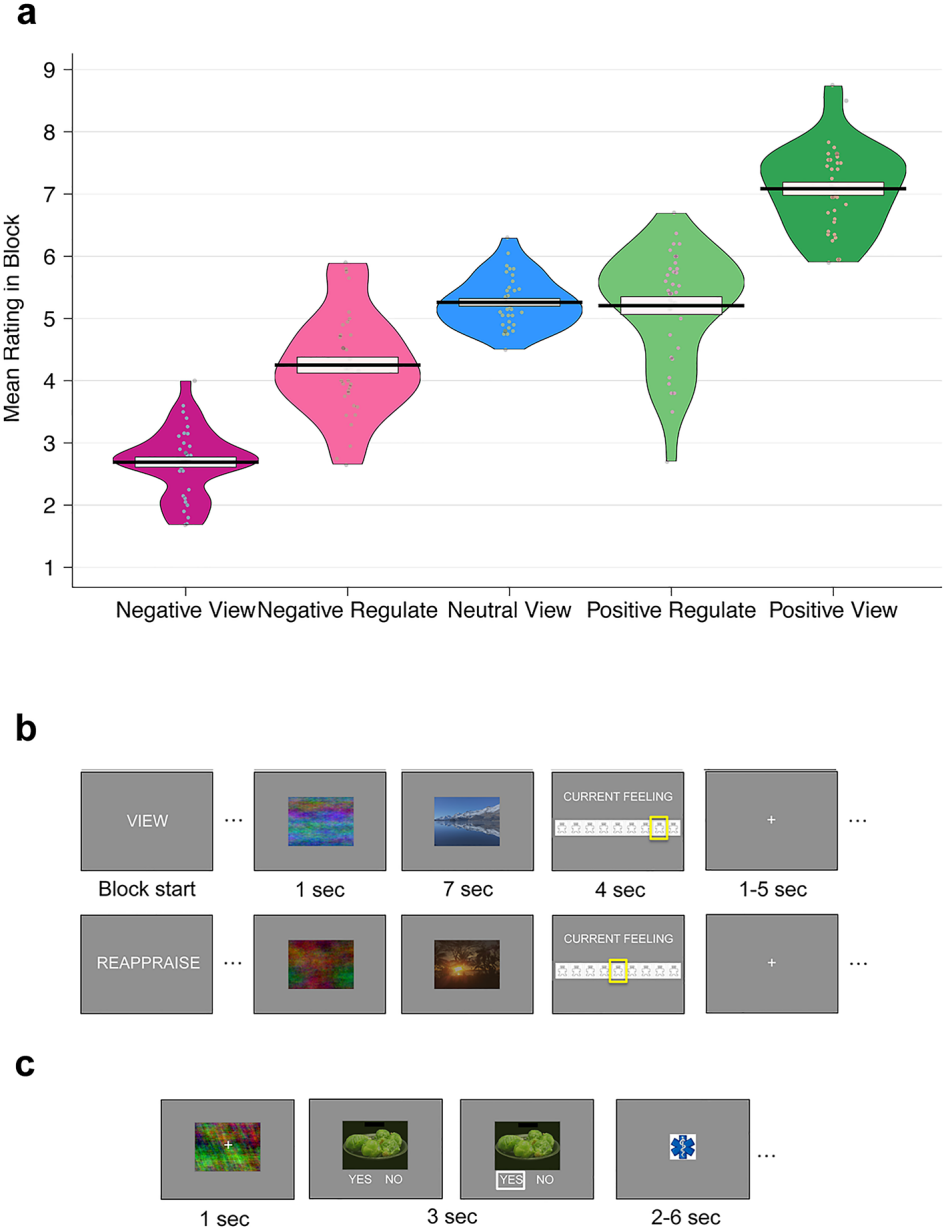
Self-regulation tasks and behaviour. (**a**) Emotion reappraisal success. On the 9-point SAM scale, the panel shows the mean emotion ratings for each block (negative view, negative reappraise, neutral view, positive reappraise, and positive view blocks). The black solid line indicates the group mean and the box its standard error. Each grey dot represents the mean ratings from one participant. Participants successfully reappraised both negative and positive images, shifting their feelings in both cases towards a neutral state. (**b**) Emotion reappraisal task. Participants saw positive, neutral and negative stimuli from the International Affective Picture System (IAPS). For display purposes, we replaced the IAPS stimuli by unrelated landscape photos here. In each block, participants either viewed the images without changing their emotional response (“view”) or reappraised the scene to render their feelings more neutral (“reappraise”). To allow the pupil to adapt to brightness and contrast, a phase-scrambled version of the stimulus was displayed for 1 s before the image was revealed. Participants then viewed or reappraised the scene for 7 s before they rated their current feeling on a Self-Assessment-Manikin (SAM) scale (for details see “Materials and methods”). A jittered inter trial interval of 1–5 s separated the trials. (**c**) Dietary health challenge task. Participants made 100 choices indicating whether they wanted to eat the displayed food at the end of the study. A phase-scrambled adaptation stimulus was presented for 1 s before the food image was revealed and participants had 3 s to decide by pressing the left or right button for selecting the answers “yes” or “no”. A white frame highlighted the answer for 0.1 s when it was logged. Trials were separated by a 2–6 s (jittered) inter-trial interval.

The fMRI part of this experiment was used to test a separate hypothesis that is reported in Maier and Hare^73^. For completeness and easier accessibility of the current paper, we summarize the behavioural results on emotional stimulus reappraisal again here. After viewing or reappraising affective pictures, participants rated their current affective state (Fig. 1a, Supplementary Table 1). On the scale from 1 (very negative) to 9 (very positive), ratings after reappraising negative content shifted closer to neutral (mean negative reappraise rating = 4.25 ± 0.81 SD) and were distinctly more positive than ratings after viewing negative content as assessed by the Bayesian equivalent of a paired *t* test (BEST^74^) that estimated the Posterior Probability of Negative Reappraise being greater than Negative View ratings (PP(Negative Reappraise > Negative View Ratings)) > 0.9999 (BF > 10,000, all participants on average shifted their ratings). Likewise, current emotions were rated more neutrally after reappraising the positive stimuli (mean positive reappraise rating = 5.21 ± 0.9 SD). Ratings after positive reappraisal were also clearly more neutral than when participants viewed positive content and let their emotional response occur naturally (PP(Positive Regulate < Positive View Ratings) > 0.9999; BF > 10,000, all participants on average shifted their ratings). Thus, participants successfully regulated their emotions by reappraising the stimulus content.

### Pupil during emotion regulation

**Figure 2:**
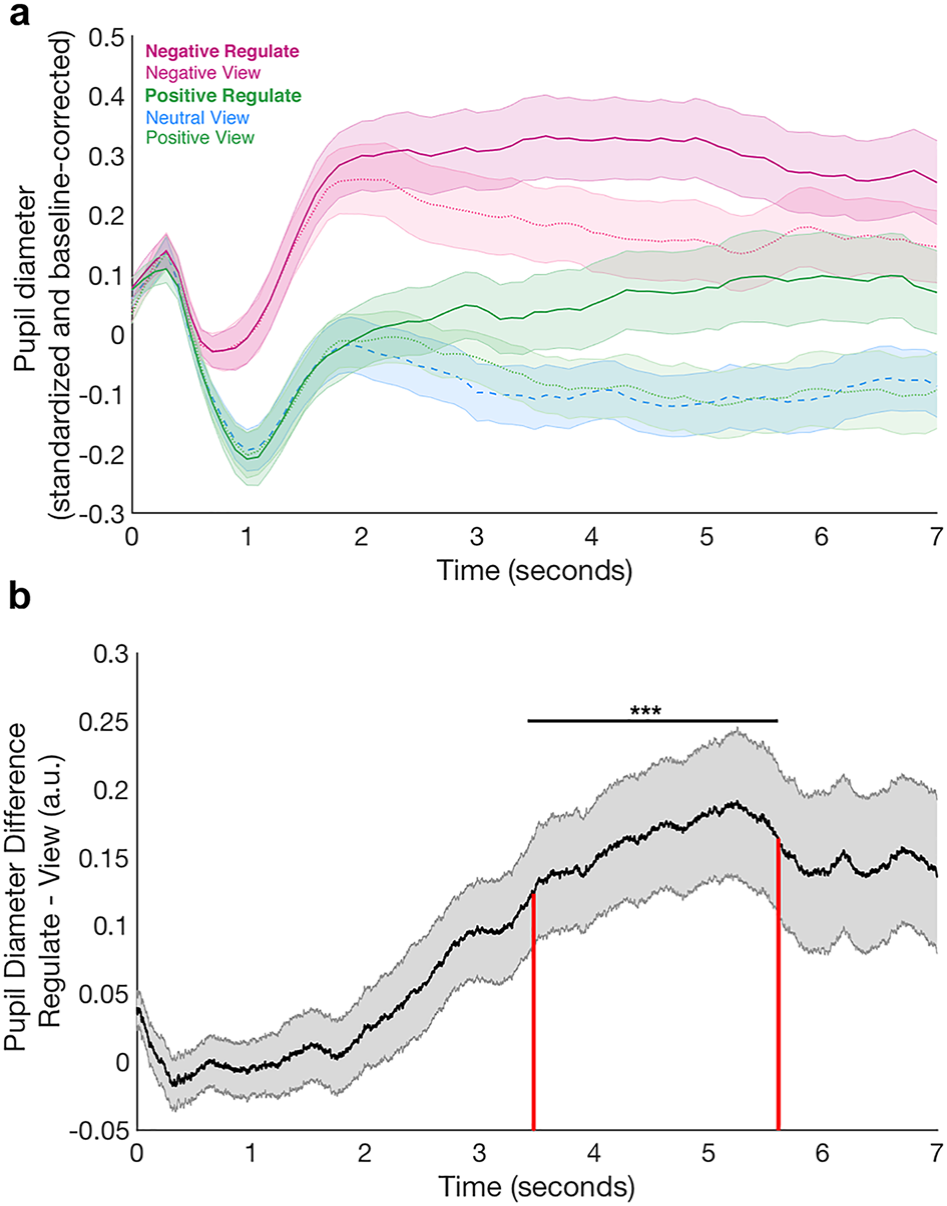
Pupil results. (**a**) Mean pupil dilation during the 7-s regulate (saturated colours) and view periods (lighter colours). The signal across the whole emotion task was z-scored within-participant and baseline-corrected on each trial for the pupil size in the 500 ms prior to the regulation period (during which the phase-scrambled adaptation version of the stimulus was displayed, see “Materials and methods”). The mean pupil dilation was calculated over all 20 trials in each block for each participant and then averaged across the group. The shaded areas indicate the standard error of the mean across participants. Lines denote the mean pupil dilation at each time point. The dotted blue line represents the neutral view condition for comparison. (**b**) Pupil Diameter Difference between Regulation and View Trials. We collapsed over positive and negative blocks in order to test for a valence-independent regulation signal across all participants. The mean z-scored pupil dilation for positive and negative view trials was subtracted from the mean z-scored dilation during positive and negative reappraise trials for each participant. A cluster-based permutation test indicated that between 3.4 and 5.6 s of the reappraisal/view period, the pupil dilation in reappraisal blocks was larger than during viewing blocks (p < 0.001; indicated by the black horizontal line and stars). For each participant, the mean of the Reappraisal – View difference in the pupil dilation signal during this period (marked by the red vertical lines in the plot) determines the Pupil Dilation Index. The shaded areas indicate the standard error of the mean across participants. The black graph denotes the mean pupil dilation at each time point.

To test our hypothesis that regulation signals are reflected in the pupil dilation signature, we compared the baseline-corrected pupil dilation time course during reappraisal with the pupil dilation time course in the view condition. Positive and negative reappraisal showed similar pupil dilation differences over time: regulation was characterized by an increase in pupil diameter from around 2 s onwards, compared to the viewing conditions, in which the pupil started constricting on average again at this time (Fig. 2a). Hence the additional pupil dilation during reappraisal may indicate participants are engaging in the regulation process, regardless of valence.

In order to rigorously test during which time period pupil dilation signatures for regulation and viewing differed without having to predefine a window for the analysis, we used a cluster-based permutation *t* test. This method identified adjacent time bins in the pupil time course that significantly differed between the regulation and view conditions. To characterize a valence-independent regulation signature, we collapsed the data across positive and negative regulation periods and generated a contrast that identified changes due to regulation regardless of valence. For each participant, we averaged the pupil time course during positive and negative reappraisal and subtracted the average pupil time course during positive and negative view conditions. We then performed a non-parametric cluster-based permutation *t* test on this contrast. This analysis confirmed that the pupil was dilated more during regulation compared to the viewing periods: within the stimulus presentation time window of 7 s, the test indicated that between 3.4 and 5.6 s after stimulus onset the pupil was significantly more dilated during regulation than during viewing (Fig. 2b; mean PDI across the group = 0.16 ± 0.003, p < 0.001, maximum T-value during this time = 3.74). We thus identified the time period during which we could reliably measure a regulation signal in the pupil time course.

As we had subtracted out all activity related to viewing pictures of equivalent emotional valence and arousal and had controlled the physical stimulus properties in the analysis (see “Materials and methods”), we reasoned that during this time window, the pupil dilation difference between the reappraise and view condition would most likely be driven by cognitive processes supporting regulation (or concomitant increases in arousal). Notably, we can exclude the possibility that the signal was merely driven by the average emotional arousal level usually induced by these stimuli, because we had designed the blocks such that stimuli shown in the regulation and view conditions were equated for their average arousal ratings, and thus the average emotional arousal pertaining to the stimuli was individually controlled for when generating the contrast. We can also exclude any other properties of the stimulus sets driving this effect, because the stimulus sets in each condition featured equally often on both sides of the subtraction contrast. We thus calculated the mean difference of the Reappraise > View contrast during the significant time window in order to quantify individual differences in regulation engagement and used this measure as Pupil Dilation Index (PDI) of regulation.

### Pupil predicts regulation success

**Figure 3:**
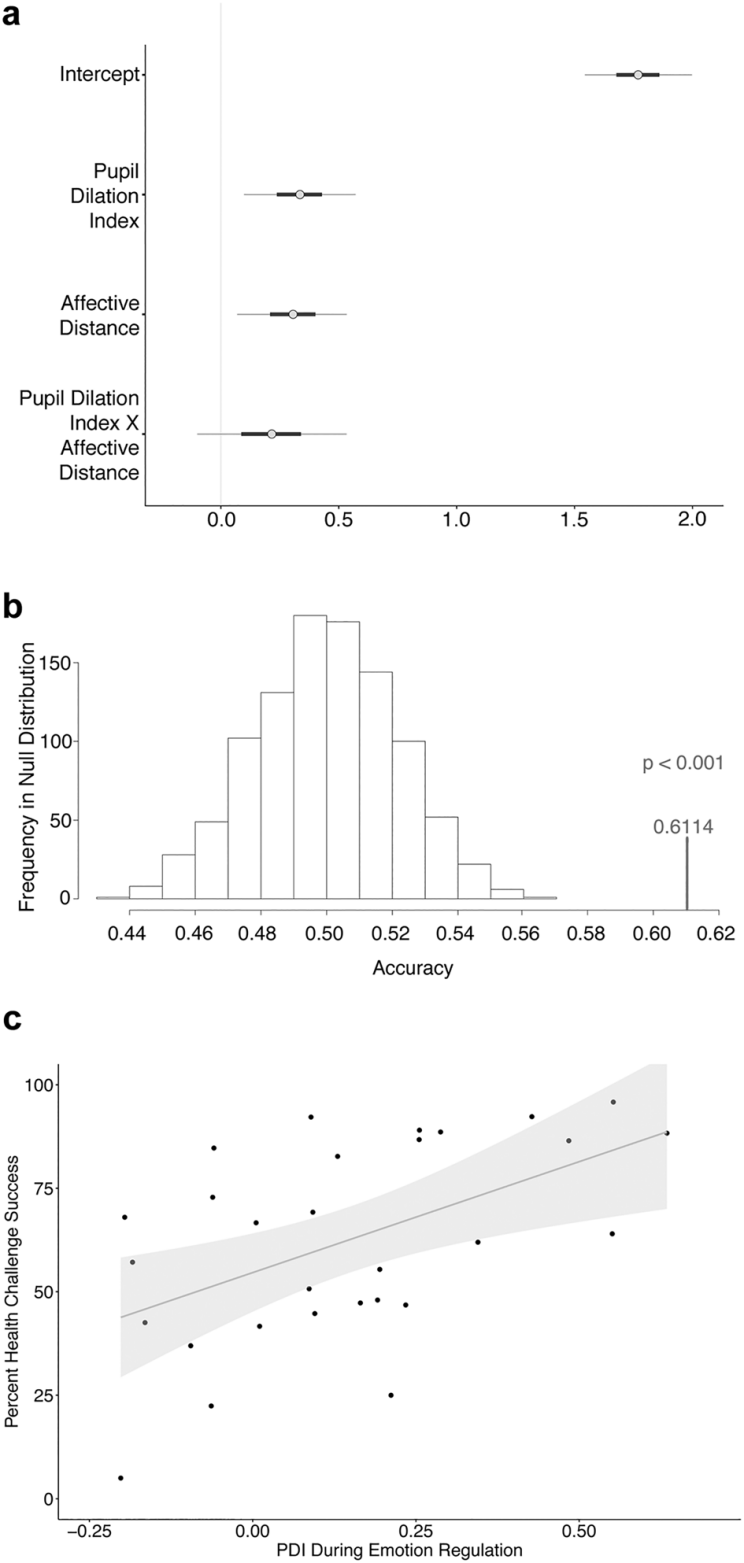
Predictive and convergent validity of the Pupil Dilation Index for self-regulation success. (**a**) Bayesian linear regression of self-regulation success using the model given in Eq. (3a). Pupil Dilation Index denotes the coefficient estimating the influence of regulation-related arousal components. Affective Distance denotes the coefficient estimating the influence of the mean absolute difference of the view rating from neutral (middle of the rating scale) that can be interpreted as a proxy for regulation difficulty. Regulation success increased both with greater Pupil Dilation Index and Affective Distance. The plot shows the mean beta estimates (grey dots) as well as the range of coefficients within the 90% credible interval) that is shown by the light grey horizontal bars (thick horizontal bars = 50% credible interval). (**b**) Predicting regulation success out-of-sample. The results from the model in Eq. (3a) were cross-validated with a Leave-2-Participants-Out approach. Based on data from N-2 participants, we predicted which of the two left-out individuals regulated more successfully. The model predicted with 61% accuracy significantly above chance (p < 0.001). (**c**) Convergent validity of the Pupil Dilation Index. The Pupil Dilation Index (PDI) that we measured during emotion regulation also shows convergent validity across different types of self-control tasks: a higher PDI value was associated with higher health challenge success scores within-individual in the dietary health challenge task that individuals completed in the same experimental session (r = 0.51, posterior probability rho > 0 = 0.9995), explaining 26% of the variance (R-squared = 0.26) in dietary health challenge success. The grey shaded area signifies the 95% confidence interval.

Using this Pupil Dilation Index, we performed a Bayesian linear regression (Eq. 3a) to account for variations in emotion regulation success between individuals. As task difficulty may impact on regulation success, the regression included the average difficulty each participant faced. As a proxy for difficulty, we calculated a measure for the affective distance of the stimulus from neutral. The absolute distance between the post-task view ratings for the regulated images and the neutral point of the rating scale constitutes the affective distance. The mean of the affective distance over all trials generates one index of average task difficulty for each participant. Both the Pupil Dilation Index (Fig. 3a, Table 1; beta = 0.34 ± 0.14 Standard Deviation (SD), 95% Credible Interval (CI) = [0.06; 0.61]) and affective distance (beta = 0.30 ± 0.14 SD, 95% CI = [0.02; 0.58]) explained substantial portions of variance in regulation success across participants. Greater central arousal activity during regulation was thus related to greater reappraisal success, above and beyond the effects of affective distance.

**Table 1:** Emotion regulation success explained by Pupil Dilation Index and affective distance.

	Beta estimate	Standard deviation	95% Credible interval
**(a)**			
(Intercept)	1.77	0.14	[1.50; 2.03]
Pupil dilation index	0.34	0.14	[0.06; 0.61]
Affective distance	0.30	0.14	[0.02; 0.58]
Pupil dilation index × Affective distance	0.21	0.19	[− 0.15; 0.58]
Bayes factor	1.83		
**(b)**			
(Intercept)	1.83	0.20	[1.43; 2.24]
Pupil Dilation Index	0.33	0.16	[0.01; 0.64]
Affective distance	0.28	0.15	[− 0.01; 0.57]
Age	0.07	0.15	[− 0.23; 0.35]
Task order	− 0.13	0.29	[− 0.71; 0.44]
Pupil dilation index × Affective distance	0.20	0.20	[− 0.18; 0.61]
Bayes factor	0.54		

(a) Results from the Bayesian Linear Regression specified in Eq. (3a). Regulation success (measured on the 9-point SAM scale) was modelled by the mean-centred and standardized coefficients for Pupil Dilation Index (measured as mean difference in the pupil dilation curve for the Regulate > View contrast in the significant regulation time between 3.4 and 5.6 s) and Affective Distance (measured as the absolute value of the distance of the view rating from the neutral valence on the SAM scale). Pupil Dilation Index and Affective Distance were interacted, to test whether it might be easier or harder to reappraise with smaller or greater affective distances.

(b) Augmenting the model (Eq. 3b) by the control regressors for Age and Task Order shows that the explanation of regulation success by PDI is robust to controlling for the age and the order in which participants completed the emotion regulation and dietary health challenge tasks.

Model fits are given as the population level mean of the posterior distribution ± standard deviation (SD) and the 95% credible interval. Bayes Factors are given for a comparison to an intercept-only model.

In order to check the robustness of this result, we ran a control analysis augmenting the model by the age of the participants and the order in which they performed the emotion regulation and dietary health challenge tasks (Eq. 3b). When accounting for these alternative explanations of regulation success scores, the Pupil Dilation Index still explained signification portions of the variation in reappraisal success (PDI coef. = 0.33 ± 0.16 SD, 95% CI = [0.01; 0.64]), whereas none of the control regressors did (Table 1b). In order to rule out that the result was driven by potential reappraisal spillover effects on the view ratings that were taken after the images had been first reappraised, we also ran a control analysis for the model in Eq. (3a) using block-wise measures for reappraisal success and affective distance that we constructed based on the equivalent stimuli from the “view” condition and found that our conclusions remained unchanged (see Supplementary Methods, Results and Discussion; Supplementary Figure 2 and Supplementary Table 2). Moreover, our results were also not influenced by the number of eye-movements per condition: the number of saccades was not different between the regulate and view conditions (see Supplementary Methods and Results, Supplementary Table 3).

To test the predictive validity of the Pupil Dilation Index, we first conducted a leave-two-participants-out analysis, in which we fit the model specified in Eq. (3a) to a training set of all combinations of participants leaving out two participants as a test set. On each iteration we predicted based on the estimates of the training set for the two test participants which of two individuals would regulate more successfully. We successfully predicted with 61.14% accuracy out-of-sample which of the test participants was better at regulating emotion responses (Fig. 3b). A permutation test revealed that this accuracy would have occurred on less than 1 in 1000 occasions if the prediction were based on randomly labelled instead of true data (p < 0.001).

### Convergent validity across self-control domains

In order to test the convergent validity of the Pupil Dilation Index across self-control domains, we performed a Bayesian rank correlation analysis between the Pupil Dilation Index from the emotion regulation task and the health challenge success levels that the same individuals achieved in a separate dietary health challenge task completed on the same day. A higher Pupil Dilation Index during emotion regulation was associated with higher dietary health challenge success levels in this separate task (Spearman’s rho = 0.51, 95% Credible Interval = [0.232; 0.759], posterior probability (rho > 0) = 0.9995; Fig. 3c; R-squared = 0.26; Bayes Factor (BF) = 28.13). Critically, this association was robust to the order of conducting the self-regulation tasks (Eq. 4). Task order did not explain variation in dietary health challenge success levels beyond the Pupil Dilation Index (Table 2).

**Table 2:** Dietary health challenge success by Pupil Dilation Index and task order.

Population-level effects	Beta estimate	Standard deviation	95% Credible interval
(Intercept)	61.96	5.81	[50.88; 73.89]
Pupil Dilation Index	12.47	4.40	[3.39; 21.08]
Task order	1.39	8.48	[− 15.58; 17.81]
Bayes factor	4.07		

Dietary health challenge success modelled with a Bayesian linear regression as a function of Pupil Dilation Index and task order (Eq. 4): the individual dietary health challenge success level (measured over all challenging trials in percentage points) is explained by the standardized and mean-centred Pupil Dilation Index (PDI). Compared to the average level of PDI in the group, an increase of 1 standard deviation (SD) in pupil dilation explains an additional 12.47% of dietary health challenge success on top of the 61.96% health challenge success that an individual with average PDI would show. The analysis is controlling for a factor representing Task Order (emotion regulation or dietary choice task first). Task order does not explain variation in dietary health challenge success levels (95% credible interval for the estimate includes zero).

Model fits are given as the population level mean of the posterior distribution ± standard deviation (SD) and the 95% credible interval. The Bayes factor is given for a comparison of the model against an intercept-only model.

The link of the Pupil Dilation Index to overall success levels in both emotion regulation and dietary health challenges suggests that the Pupil Dilation Index captures a more general process underlying self-regulation success in both task domains. Its predictive power with 26% explained variance across tasks (R-squared = 0.26) and decisive Bayes Factor of 28 provides converging evidence that the Pupil Dilation Index measured an important correlate of self-control success.

## Discussion

We find that the pupil dilation difference between reappraising and viewing emotional stimuli predicts the degree to which individuals are able to render their feelings more neutral. We demonstrate that this is tied to the individual level of regulation-related arousal, indexed by pupil dilation, and independent from the estimated effects of task difficulty that we quantify via affective distance (Eq. 3a). This finding was also independent of the participants’ age and the order in which the self-control tasks were performed (Eq. 3b). In order to ensure that this relationship was not merely driven by potential spillover of reappraisal on the subsequently collected ratings under view conditions^75^, we performed a control analysis with measures constructed based on the “view” condition. Note that any reappraisal spillover would have weakened the evidence instead of supporting our conclusions. In the control analysis, we found the same benefit of higher regulation-associated arousal to emotion regulation success (see Supplementary Methods and Results).

It also speaks against an explanation of the regulation success purely by an experimenter demand effect that we identified a relationship between the regulation success and a physiological measure for which voluntary manipulations would have been easily detected. This association should not hold if all variance in the reported emotion after regulation were explained by the desire to conform to the task instructions. As a further piece of evidence that the emotion task and pupil analyses worked as planned, our results for viewing emotional stimuli without regulating replicated previous findings^21,31,41,59,63^.

Importantly, we isolated regulation-related signals in the pupil dilation time course. The experimental design required to compute the Pupil Dilation Index is rather simple and can be repeated with a broad range of stimuli for other cognitive experiments that go beyond the realm of emotion control. As we demonstrate in our paper, potential interpretative caveats can be addressed upfront through a combination of experimental design features that allow to construct the Pupil Dilation Index (see Supplementary Discussion).

Our results indicate that both the Pupil Dilation Index and affective distance independently explain variance in how successfully individuals render their feelings more neutral, pointing towards separable factors that influence self-control success. Despite subtracting out the signal related to the average emotional arousal properties of the stimuli through the Reappraise > View contrast, we still observed pupil dilation, indicating increased regulatory arousal during the reappraisal process. If the pupil dilation signature during the regulation condition was purely explained by the arousal caused by viewing the content of the image, the pupil diameter should decrease once regulation sets in. In our regulation condition, however, pupil dilation stayed elevated for a prolonged period, suggesting that regulation still further engaged the pupil-linked arousal system.

Nevertheless, emotional responses and emotion regulation are likely to be bidirectional and recursive, such that the emotional arousal component in the pupil dilation time-course may in fact change in response to the regulation, and thus differ between the mere viewing- and the regulation-condition because the emotional arousal is also likely to decrease when the emotional response to the stimulus is rendered more neutral during reappraisal. It is possible that individual regulation success may depend on the regulatory effort exerted (as a proxy indicated by pupil dilation increase) or the resulting emotional arousal decrease (associated with a pupil dilation decrease) or a combination of both. We here use the Pupil Dilation Index as a proxy for the extent to which cognitive control processes are recruited during emotion regulation and may link to its success. In this regard, it is worth noting that we made a rather conservative assumption when we attempted to measure the regulatory arousal via pupil dilation. The Pupil Dilation Index of regulatory arousal (Regulate > View) is a conservative measure, because we subtract the emotional arousal component of the pupil time course measured in the equivalent view block, i.e. as if no emotional arousal reduction had taken place. In our data, the observed increase in pupil dilation during regulation will most likely originate from processes associated with regulation, because post-trial ratings indicate that participants were successfully decreasing their emotional arousal in all but a few trials. The simplifying assumption to subtract out a certain average level of emotional arousal regardless of the dynamics of the regulation process is a limitation in the sense that our design is unable to quantify precisely how large a likely decrease in emotional arousal during the ongoing dynamic regulation process is. However, our measure of interest was not emotional arousal, but the arousal component indicative of regulatory cognitive processing. Our main claim that the Pupil Dilation Index may serve as a proxy of cognitive control mechanisms that generalizes across cognitive control task domains thus remains unaffected.

The most important finding of this study was that the Pupil Dilation Index, a proxy for the central arousal state, quantified and predicted how well participants applied cognitive control across tasks. We observed in this study that a higher regulatory arousal response not only predicted emotion regulation success, but also explained individual differences in another task domain requiring cognitive control, namely dietary self-control. Yet we are not proposing a directional relationship: It may either be the case that higher arousal instigates more control, or, vice versa, that during cognitive control, additional arousal is recruited to fine-tune control processes.

Beyond the well-established finding in the literature that pupil dilation increases with more taxing cognitive control tasks^66,68^, we show here that one can use this proxy to index cognitive control success within and across task domains. One potential mechanistic explanation that should be tested in future studies was proposed by Verguts^76^: arousal responses may be triggered downstream through projections of the medial frontal cortex to the locus coeruleus^53^ when cognitive control is executed and the medial frontal cortex tries to orchestrate other cortical regions. Notably, several regions that are important parts of networks supporting cognitive control (e.g., anterior cingulate cortex and medial frontal cortex) are also in another function important hubs in the central autonomic network^77–79^. In case of a re-purposing of these regions as contributors to cognitive control, it may still be the case that those central autonomic network regions are concomitantly tapping the noradrenergic arousal response, triggering downstream effects that can be quantified by pupil dilation. In other words, our Pupil Dilation Index may be a good proxy for the activity and responsivity of these shared common regulation mechanisms that underpin different cognitive control tasks. As to why this metabolically costly mechanism is recruited^80,81^, multiple theories propose that the concomitant arousal response may additionally serve to facilitate cognitive control as we outline below.

One reason for our observations may be that participants engage the arousal system in order to regulate. This is in line with reports of higher pupil dilation readouts during similar tasks^21,63^ that show a similar pattern of increasing sympathetic signalling within the regulation period. Pupil dilation is a natural candidate to measure such arousal in service of cognitive control^51,82–89^, because it is tightly coupled to activity in the locus coeruleus that releases noradrenaline^52,55,90–93^.

Our data are consistent with different mechanistic theories on the relationship between arousal and cognitive control processes. However, our experiment was not designed to distinguish between potential mechanisms (such as effort, attention, or working memory processes) that may all be invigorated by arousal and benefit cognitive control, and all would yield pupil signatures similar to the one we observed.

For example, Varazzani, et al.^27^ suggested that noradrenaline release of the LC may serve to increase motivation to exert effort for an encountered challenge at the time when an energetically costly action is performed. Moreover, the “adaptive gain” theory^53^ postulated more generally that phasic noradrenaline release by the locus coeruleus helps to facilitate behaviours that optimize task performance^94^. Mather and colleagues have suggested in their GANE theory that arousal may lead to norepinephrine release that facilitates selective attention^95^ and memory consolidation^50^. Similarly, Verguts and Notebaert^56^ have proposed in their “adaptation by binding” theory that arousal enhances cognitive control by facilitating communication between task-relevant cortical areas and increasing online Hebbian learning that binds together representations that are activated together. Attentional focus and modification of associations in memory are both relevant to emotion regulation via reappraising the content of the pictures^96^. In line with the “GANE” and “adaptation by binding” theories, one might speculate that the impact of arousal on memory processes may contribute to reappraisal success: if old associations that have been activated were more malleable under arousal, the increase in regulatory arousal during the reappraisal process may foster online learning of new associations (for a review on pupillometry measures of memory formation and retrieval see Papesh and Goldinger^97^). Moreover, this process may profit from better attentional focus on the aspects of the stimulus that are to be re-framed.

Regardless which of these mechanistic explanations contributes to more successful reappraisal, it is noteworthy that we seem to tap into a more general process that holds beyond the reappraisal of emotional stimuli. The Pupil Dilation Index has predictive validity, as evidenced by the out-of-sample prediction of emotion regulation success. We also demonstrate its convergent validity: it is associated with success in a dietary health challenge task. This strongly suggests that the Pupil Dilation Index captures a more general process supporting successful regulation across multiple task types and may serve as a promising measure in future investigations of self-control mechanisms in health and disease.

At this point, we can only speculate which aspect of emotion regulation the Pupil Dilation Index picks up that links to the success in both tasks. Fernandez et al.^71^ proposed that emotion regulation might form an own domain according to the Research Domain Criteria (RDoC) that emerges when other RDoCs are combined (negative valence systems, positive valence systems, cognitive systems, social processes, and arousal and regulatory systems). Through our experimental approach, we narrow down the common contributors to self-control success across domains that we indexed with the pupil measure to the RDoC components of cognitive systems, and arousal and regulatory systems. We demonstrate a link to self-regulation success across domains specifically through an autonomic nervous system measure used as a proxy for central arousal levels (pupil dilation). We also found in prior work that other indices of the flexibility of the reaction of the autonomic nervous system, heart rate and heart rate variability predicted anxiety symptoms during chronic stress^98^ and health challenge success in dietary choices^99^ respectively. We may therefore cautiously speculate that the reactivity of arousal systems might play a role in self-control success through a facilitation mechanism in which dynamic changes in arousal support or tune cognitive processes for self-control. Interestingly, our results also suggest that a greater dynamic range for increasing central arousal may help to boost emotion and other forms of cognitive control. This may bear important implications for the treatment of patients with ADHD and autism spectrum disorders, for whom high arousal at baseline but lower reactivity to evoked stimuli are observed in tasks that test sustained and selective attention. In ADHD, pupil dilation in response to stimuli is reported to be lower^15^, but at baseline alertness may be tonically increased (see^14,16,17^), similarly as in autism spectrum disorders^18^.

Our study also presents several advances regarding the study of emotion regulation in general. Our approach measures regulation effects in pupil dilation in a continuous fashion, which allowed capturing the actual onset of regulation. The two emotion regulation strategies, reappraisal and distraction, have been associated with distinct patterns of visual attention and differing levels of cognitive demand^33,59^. Such pupil dilation effects may increase with age^32^. Our pupil data corroborate prior work regarding the time course differences between emotion regulation versus mere viewing the emotion inducing stimulus. Similarly to our data, previous studies also reported increases in pupil dilation after 2 s of stimulus presentation between the regulatory pupil and the mere viewing time course^33,59^. The congruence of these results, given similar stimulus presentation procedures, therefore indicate that the onset of regulatory arousal for the reappraisal strategy seems relatively stable across individuals and studies, with a start time approximately 2 s after the emotional stimulus is presented.

A potential application of the Pupil Dilation Index in affective disorders may be to measure the progress an individual makes in finding and applying more effective and appropriate strategies to regulate their reactions to emotionally salient events or thoughts^1,100^. We believe that this may foster further lines of research, for instance on how individuals flexibly use different types of emotion regulation strategies^101–103^. For instance, there is ample prior evidence that reappraisal is demanding and energy consuming^104–107^ and several physiological indicators such as Skin Conductance Response (SCR) and Electromyography (EMG) have corroborated this notion^105,108–110^. Compared to SCR and EMG, the Pupil Dilation Index is much easier to apply and may be used to investigate on an individual basis how well a regulation strategy may work and specifically how effortful a given strategy may be for each individual^111^.

Our results hold the potential to further advance our understanding about clinical conditions in affective disorders. A number of recent studies have found lower pupil dilation during various tasks involving emotion perception and/or regulation in disorders in which emotion regulation impairments are typically observed, such as depression^112,113^ and anxiety disorders^114,115^. Recently, Grueschow, et al.^52^ related the noradrenergic responsivity of the locus coeruleus and concomitant pupil dilation responses during an emotional conflict resolution task to anxiety and depression symptoms induced via a prolonged occupational stressor. Moreover, another recent imaging study indicated a crucial role of the noradrenergic LC-NE in cognitive control and the individual level of regulatory behaviour^51^.

The presented Pupil Dilation Index (PDI) as a proxy for LC-NE activity may provide a neurophysiological measure to quantify the ability and readiness to deploy effortful cognitive control. The PDI may inform for example treatment decisions for depression in addition to self-report and structured interview, as longer pondering time whether to spend effort has recently been shown to predict increased relapse risk for patients with remitted depression^116^. Probing this result in future studies with the Pupil Dilation Index, we might hence expect depressive patients to show a later onset of regulation compared to healthy controls. This may contribute to a lower regulation success overall, because too late onset of reappraisal may render the strategy less successful^117^. Thus the Pupil Dilation Index may be used to predict and assess treatment outcome prior to pharmacological or cognitive therapy. The presented methodology paves the way for future studies that may use the PDI to monitor self-control capacity to assess the most efficient and most effective regulation strategy on an individual basis. In addition, future studies should also determine whether the PDI indicates which regulatory strategy may be more difficult to engage in and weigh the more demanding effort with the potential regulatory success that could be achieved with alternative strategies. Importantly, the demonstration that the PDI holds predictive power not only within but also across task domains suggests that it may be a useful index of general self-regulation capacity.

In addition, our approach might help to assess individual progress in applying trained strategies to reduce for example cravings for foods or drugs of abuse. In the future, our results may be relevant to diagnosis, treatment and intervention in psychiatric disorders and psychosomatic medicine.

In sum, we have presented a combination of innovative approaches that advance both basic and applied research on emotion regulation, and our understanding of self-control and cognitive control mechanisms more generally. We believe that pupillometry can ideally leverage modern technological advances in data acquisition and analysis. Modern eye-trackers are highly mobile and could be used at bedside or in workplace settings. In addition, they can easily be used with children and the elderly population. Data acquisition is comparatively cheap, and adjustment of stimulus material and artefact correction with modern computational tools may yield better data quality than other typical readouts of the autonomic nervous system such as heart-rate or skin conductance. Measuring pupil dilation is unobtrusive, and it cannot be manipulated covertly by the participant without direct pupil feedback. Combining these advantages enables assessing spontaneous changes in arousal without asking individuals for their self-report. Our Pupil Dilation Index may provide means to replace or at least further inform self-reports. The results presented here, given replication may help to avoid self-report biases in the future. For clinicians, this facilitates data collection when individuals have difficulties with regard to interoception or introspection, and for basic science, it yields neurophysiological readouts to develop and to test theories.

Taken together, our physiological and behavioural data across different types of self-control domains suggest that a common arousal-based facilitation mechanism contributes to individual differences in human self-control in complex tasks. We present evidence that this arousal-based facilitation of self-control generalizes across emotional valences, and across self-control task domains. Finally, the Pupil Dilation Index we describe in this work bears important advantages that may enhance the toolkit of research in the affective sciences, cognitive control and self-control.

## Materials and methods

### Participants

Data were acquired from 43 healthy adults. Data of thirty-four participants (20 female; mean age = 22.59 ± 2.23 SD years) were included in the pupil analyses (all exclusion decisions were made based on a priori criteria that are well established in our laboratory and before analysing any data). Seven participants did not complete the emotion reappraisal paradigm with sufficient data quality: five fell asleep during longer stretches of the task (detected by the eye-tracker), one deliberately closed the eyes when negative stimuli were displayed, and one reported experiencing pain due to head positioning during the task. We reasoned that this participant who reported his discomfort only after the study likely engaged in constant self-control that would interfere with our analyses. These participants were also excluded from the analyses in the companion paper. Pupil data could not be evaluated for two additional datasets because the eye-tracker did not record the start of the experiment correctly. All participants were German native speakers, and the experiment was conducted in German to ensure that participants were well able to follow the instructions and paradigm. Screening assured that participants were not depressed (Beck Depression Inventory (BDI) I^118^, German validated version by Hautzinger, et al.^119^) or emotion blind (Toronto Alexithymia Scale, TAS^120^, German validated version by Franz et al.^121^), because both conditions have been associated with altered emotion perception. Participants included in the pupil analyses on average scored 4.1 ± 2.8 SD on the BDI (cutoff for mild depression: 10). On the TAS, the mean score was 38.1 ± 8 SD (potential alexithymia: 52, cutoff: 61). With respect to the dietary health challenge task, screening assured that all participants were interested in maintaining a healthy diet, but also reported to like and consume snack foods and sweets at least on two occasions per week so that meaningful self-control challenges could be created.

All methods and experimental procedures were carried out in accordance with the Declaration of Helsinki. All experimental protocols were approved by the Ethics Committee of the Canton of Zurich, Switzerland. All participants were of legal age and provided written informed consent at the day of the experiment, prior to experimental measurements.

### Procedure emotion task

Emotion stimuli were selected from the International Affective Picture Set^122^. The experimenter used the instruction detailed in Lang, et al.^122^ to introduce the 9-point Self-Assessment Manikin (SAM) Scale^123^. Briefly, participants rated their current emotion after each trial with a version of the SAM scale validated by Suk^124^ that displayed 9 Manikins for the valence. The most negative feeling was coded as 1, and the most positive feeling was coded as 9. The scoring direction was counterbalanced across participants by randomizing whether the most negative valence would be scored on the left or on the right side of the scale. The reappraisal task was based on Wager et al.^19^. We additionally included stimuli with positive valence, which allowed us to investigate domain-general regulation and facilitates comparisons of the emotion reappraisal paradigm with the dietary health challenge task that the same cohort of participants solved on the same day (see below). Participants practiced the reappraisal task in a standardized procedure (see Supplementary Methods) on stimuli that were not repeated in the evaluated task. In the view condition, participants were instructed to simply look at the image and let the emotional response occur as elicited by the picture. Participants were asked not to modulate their emotional response, providing us with pupil data of unaltered positive and negative picture viewing (Fig. 2a, dotted lines). In the reappraisal condition, participants were asked to try and come up with an alternative scenario accounting for the observed scene, such that the evoked emotional response moved more towards a neutral state.

Blocks of 20 trials of either condition were cued by displaying the words “view” or “reappraise” for 1 s centrally on the screen (Fig. 1b). At the beginning of each trial, participants saw a phase-scrambled version of the stimulus image for 1 s that allowed the pupil to adapt to low-level stimulus properties. The emotion content version of the picture was then displayed for 7 s as in Wager et al.^19^.

Because of the adaptation period in our design, we were able to baseline-correct the signal on each trial based on the physical features of the actual stimulus displayed subsequently. This allowed us to interpret changes in pupil dilation during the view period as being primarily driven by the emotional content of the stimulus, while during reappraisal trials regulation-related pupil responses should be observed in addition to the emotional arousal induced by the stimulus content. During the 7 s stimulus presentation, participants had to either view the image without regulating their emotional arousal or reappraise their emotional responses to make them more neutral according to the trained procedure. To remind them of the currently relevant task condition, a short cue (“V” for view or “R” for “reappraise”) replaced the fixation cross overlaid on the stimulus. Participants were asked to fixate these central markers, which were present throughout the trials, to minimize saccadic influences on pupil dilation measures. After the stimulus presentation, participants rated their current emotional response on the 9-point SAM valence scale (within 4 s). A jittered inter-trial interval (uniformly sampled from 1 to 5 s) separated the trials.

Block types (Reappraise Positive, Reappraise Negative, View Positive, View Negative, View Neutral) were presented in five pseudo-randomized orders that ensured that valence changed after each block. Each block was followed by a 15-s break, and the task was performed in a single run. This run lasted ca. 26 min, with each block of 20 trials lasting ca. 5 min and each trial lasting ca. 15 s on average.

### Emotion regulation success measurement

To quantify reappraisal success, participants rated all 40 stimuli that had been presented in the reappraisal condition while sitting at a standard computer terminal. Participants were asked to rate the images as in the “view” condition, i.e.: rating the feeling elicited by the image without altering the emotion. We then quantified emotion regulation success: if negative images were successfully reappraised, participants should rate the image more positively than in the view condition. Therefore, negative reappraisal success was calculated according to Eq. (1):

1 $${\text{Negative}}\;{\text{reappraisal}}\;{\text{success }} = {\text{ reappraisal}}\;{\text{rating }}{-}{\text{ view}}\;{\text{rating}}$$

Vice versa for positive stimuli, the rating after successful reappraisal should be more negative than the view rating, and was thus calculated as in Eq. 2:

2 $${\text{Positive}}\;{\text{reappraisal}}\;{\text{success }} = {\text{ view}}\;{\text{rating }}{-}{\text{ reappraisal}}\;{\text{rating}}$$

In order to obtain the overall reappraisal success score for each participant, we then averaged over positive and negative reappraisal success scores.

### Dietary health challenge task

Methodological details for this task are described in a companion paper by Maier and Hare^73^. Briefly, in the dietary health challenge task (Fig. 1c), one food item was presented on each trial, and participants had 3 s to indicate whether they wanted to eat this food or nothing at the end of the study (participants had fasted 3 h prior to completing this task). Choices were customized based on the individual taste and health ratings such that in challenging choices we presented each participant with foods that they had rated (a) subjectively tastier and less healthy than neutral, or (b) healthier and less tasty than neutral. In the remaining choices, health and taste attributes were aligned. Trial types (challenge or no challenge) were randomly mixed. The dietary health challenge success level was defined as the proportion of challenging choices during which participants refused to eat tasty-unhealthy foods or accepted to eat healthy-untasty foods.

Participants completed the dietary health challenge task and emotion regulation task in single runs with 100 trials each. Tasks were acquired in counterbalanced order, with a 7-min break in between.

### Stimulus selection

The stimuli that participants viewed or reappraised in the emotion regulation task were selected from the International Affective Picture System (IAPS) database (for a full list please see the Supplementary Methods). These photographs have been widely used in emotion regulation research^19,24,125,126^ and their arousal and valence levels have been quantified and validated in large population studies^122,127^. For the current study, we selected IAPS Stimuli based on a validation study in a German-speaking sample^128^. Based on the mean ratings given by the young adults in this dataset, we identified 40 images that scored highest on positive and 40 images that scored highest on negative valence. To minimize confounds in identifying physiological correlates of the regulation process, we next aimed to equate the average arousal levels of these stimuli. For both positive and negative stimuli, we created two sets of 20 stimuli each. To control for the average arousal and valence levels, we distributed the images between the sets such that both sets in each domain scored on average the same for valence (mean negative = 2.25 ± 0.29 SD; mean positive = 7.25 ± 0.24 SD) and for arousal (mean negative: 6.99 ± 0.44 SD; mean positive: 2.86 ± 0.43 SD, based on the ratings of the sample in Grühn and Scheibe^128^). We then randomly allocated for each participant which set would be presented in the “view” and “reappraise” condition for each valence domain. We also identified 20 images that scored neutral on both valence and arousal. When selecting the images, we excluded any that showed foods (due to the dietary health challenge task performed on the same day) and replaced them with the next best-scoring image.

### Pupil data acquisition

Pupil diameter was sampled at 500 Hz using an MR-compatible EyeLink II CL v4.51 eyetracker system (SR Research Ltd). Prior to the start of the paradigm, the recording quality was tested and adjusted with a spatial 9-point calibration task, covering the full screen dimensions and assuring that tracking would not be compromised by large eye-movements. Blinks and signal loss were linearly interpolated^129–131^. To detect blinks and signal loss, we used custom software, written in Matlab, which first generated a binary vector across time, indicating signal loss with 1 and otherwise 0, identified by built-in detection techniques provided by EyeLink. We next convolved this vector using a Gaussian window (FWHM 200 ms; for an alternative approach see for example Hershman et al.^132^) and transformed it back to a box-car function using the Matlab routine ceil.m. Finally, applying diff.m on this box-car function returned the start and endpoint in time for linear blink interpolation as 1 and − 1 respectively.

Perceived brightness and stimulus contrast are low-level visual stimulus features that have previously been shown to affect pupil size^84,133–139^. To facilitate isolating pupil signals related to self-control mechanisms during reappraisal, we introduced an adaption phase before each trial that allowed us to account for low-level stimulus features. One second before the actual stimulus presentation, we displayed a phase-scrambled adaptation version of the stimulus (Fig. 1b, second screen) that did not reveal the semantic content of the scene, but allowed the pupil to adapt to contrast and brightness of the target stimulus (Fig. 1b, third screen; please see the Supplementary Methods for details of the algorithm that matched properties of the adaptation and target stimulus pairs and Supplementary Figure 1 for the close correspondence between adaptation and target stimuli).

### Pupil analyses

Our approach applies principles of time series analysis to the pupil recordings. In order to attain a common frame of reference for the pupil dilation, all pupil time courses were z-scored per participant across all trials (i.e., across the whole run). We also baseline-corrected the within-trial pupil signal by subtracting the average pupil size during the 500 ms period before stimulus onset^130^. We then constructed a subtraction contrast to isolate signal components specific to regulation that differ from pupil dilation signals associated with merely viewing emotional stimuli.

We aimed at characterizing common regulation processes during both positive and negative reappraisal and thus collapsed the data over the positive and negative valence domains. To dissociate the portions of the pupil dilation signal primarily driven by regulation, we subtracted the average pupil dilation during simple viewing of emotional stimuli inducing emotional arousal, from the pupil dilation measured during reappraising equivalent emotional content in stimuli that were on average equated for valence and arousal as described above. For convenience, we will henceforth describe this subtraction as the “Reappraise > View” contrast. By construction of these intra-individual contrasts and thereby at the same time controlling brightness between the reappraise and view conditions, we rule out confounds following the recommendations by van der Wel and van Steenbergen^68^. In line with the recommendations by Goldinger and Papesh^140^, we also baseline-corrected the signal for the tonic pupil diameter before the trial and used inter-trial intervals of at least 1 s in addition to the adaptation period. Furthermore, the Reappraise > View subtraction contrast was evaluated at the group level, and across the group, randomization ensured that both subsets of stimuli were featured equally often in the reappraise and view conditions. This entails that any stimulus-specific effects in the pupil dilation signal average out in the analysis.

To identify time periods during which the pupil dilation significantly differs between regulation and mere viewing, we performed a cluster-based permutation test following Nichols and Holmes^141^ with a cluster-forming threshold of T = 3 applied to each time bin. Briefly, we first for each participant took the mean of the reappraise time series and the mean of the view time series (collapsing across positive and negative valences) during the 7-s reappraise/view period and calculated the difference between both in order to compute the Reappraise > View contrast. We then calculated the one-sample t-statistic for each millisecond time bin in this contrast. This *t* statistic indicated for each time bin whether the signal difference during reappraising versus viewing significantly differed from zero, which allowed identifying periods of consecutive time bins that exceeded the cluster-forming threshold. These defined the sizes of the temporal clusters (i.e., number of adjacent time bins exceeding the threshold of T = 3) to test against a null distribution in the next step. The null distribution was generated by permuting the labels for each time bin within-participant for 1000 iterations (flipping the sign of each time bin randomly 1000 times). On each iteration, we then again calculated for the average time series within each participant the one-sample t-statistic for each time point and identified the time bins in the permuted t-statistic vector that exceeded the cluster-forming threshold. We thereby identified the cluster sizes of each permuted cluster and stored the largest permuted cluster in the null distribution on each iteration. Note that by storing and comparing only the largest permuted cluster (and not the mean cluster size in the null distribution), we chose a more conservative approach than Nichols and Holmes^141^. To draw inference and to calculate a p-value for the temporal clusters identified in the data, we then asked how many of the randomly generated clusters from the null distribution were larger than the cluster we observed in the data. In other words, we counted how many times a cluster of the size we observed in the true data would have occurred by chance. To calculate the equivalent of a p-value, we divided this number by 1000 (iterations) in order to judge how likely it was to obtain a cluster of this size just by chance. In our case, in the real data 2190 adjacent time bins had a *t* statistic greater than the cluster-forming threshold, and there were 0 clusters in the null distribution that had at least 2190 adjacent time bins with the *t* statistic being greater than the cluster-forming threshold. Hence the p-value would be 0/1000, so we report it as p < 0.001. We further report the maximum t-statistic occurring in this time window and the mean value of the Pupil Dilation Index and its standard error across the group to characterize the effect size. Note that we also obtained virtually identical results when using smoothed pupil data by means of applying a moving average to the pupil time series (see Supplementary Methods, Results and Supplementary Figure 3 for detail). These results indicate that future applicants of our methodology do not need to administer an additional smoothing pre-processing step. This property of our PDI may foster ease of use and simplicity in translation to multiple and diverse settings.

Pupil dilation is governed by the sympathetic branch of the autonomic nervous system (activation/arousal) and pupil constriction is governed by the parasympathetic branch (relaxation). Therefore, we assume that during the process of regulating the pupil diameter should increase and once the regulation has been successful the pupil diameter should decrease again. Arousal processes related to regulation for each individual can therefore be captured by the mean difference in the Reappraise minus View contrast during the time in which we identified significant increases in pupil dilation across all participants (between 3.4 and 5.6 s). In the following analyses, we will refer to this measure as Pupil Dilation Index (PDI).

### Influences on reappraisal success

We modelled emotion regulation success as a function of the Pupil Dilation Index and the affective distance to be regulated, using a Bayesian linear regression model described in Eq. (3a) below:

3a $${\text{RS }} = {\text{ }}\beta _{0} + {\text{ }}\beta _{1} \;{\text{PDI }} + {\text{ }}\beta _{2} \;{\text{Affective }}\;{\text{distance }} + {\text{ }}\beta _{3} \;{\text{PDI }} \times {\text{ Affective}}\;{\text{distance }} + e$$

where *PDI *(*Pupil Dilation Index*) denotes for each participant the mean difference in the pupil dilation signal during the significant regulation time window in the contrast Reappraise > View. *Affective distance* describes the average regulation distance to be covered for each participant, measured as the absolute distance of the view ratings for the regulated images from neutral. *Reappraisal Success* was measured as the average of positive and negative reappraisal success for each participant that were separately scored according to Eqs. (1) and (2). We included an *interaction term for PDI and affective distance*, because affective distance may have a simple relation to regulation success: if the starting valence is further away from neutral, more regulation success can occur as compared to smaller affective distance. However, the amount of cognitive control that is required to move to neutral could also vary depending on how far from neutral the participants starts off before regulating: in other words, it might be harder or easier to regulate, the further away from neutral the stimulus valence is. For this conceptual reason we included the interaction term when modeling the relationship of Pupil Dilation Index and affective distance to regulation success.

In order to control for potential confounds that may alternatively explain regulation success, we augmented the model as described in Eq. (3b):

3b $${\text{RS }} = {\text{ }}\beta _{0} + {\text{ }}\beta _{1} \;{\text{PDI }} + {\text{ }}\beta _{2} \;{\text{Affective}}\;{\text{distance }} + {\text{ Age }} + {\text{ Task}}\;{\text{order }} + {\text{ }}\beta _{3} \;{\text{PDI }} \times {\text{ Affective}}\;{\text{distance }} + e$$

Here, in addition to the coefficients described in Eq. (3a), the model included a standardized and mean-centred term for the age of each participant, and a factor that indicated in which order the emotion and dietary health challenge tasks were completed.

### Out-of-sample predictions

To test the ability of the main model described in Eq. (3a) to predict out of sample, we used a leave-2-samples-out approach. We generated all 561 possible combinations of training/test sets: in each training set, we estimated the model on the data of 34 participants and predicted for the test set (the two left-out participants), which of these two individuals would be more successful at regulating. We then compared the predicted to the true regulation success ranking for each of these 561 combinations and calculated the accuracy of our predictions, i.e., the proportion of true predictions out of all 561 predictions made. In order to quantify how often this accuracy would occur by chance, we generated a null distribution of prediction accuracies from 1000 iterations running the model on random training data. To this end, on each iteration, we permuted the labels for the regulation success scores of the training sets (randomly multiplying half of the training set success scores by − 1) and trained the model on these random outcome values. Based on these estimates, we then again predicted for all possible combinations of training/test sets which of two left-out participants regulated more successfully. On each iteration, we calculated the accuracy of the prediction (i.e. how many pairs of participants were correctly predicted out of all possible combinations). We thereby obtained a distribution of 1000 accuracies that would have occurred by chance. To calculate a p-value, we computed how often accuracies greater than the one that was obtained from predictions based on the true data would occur in this null distribution (i.e., by chance), and then divided this value by 1000 to account for the number of iterations.

### Predictive validity across self-control domains

In order to test the predictive validity of the Pupil Dilation Index across self-control domains, we calculated a Bayesian rank correlation between the Pupil Dilation Index from the emotion regulation task and the health challenge success level achieved by the same individuals in a separate dietary health challenge task measured on the same day. To assure that any effects were independent of the order in which tasks are performed, we modelled health challenge success controlling for task order using the Bayesian linear regression model described in Eq. (4):

4 $${\text{HCS }} = {\text{ PDI }} + {\text{ Task}}\;{\text{Order }} + e$$

where *HCS* denotes the overall health challenge success in the dietary self control task, operationalized as the percentage of challenge trials during which participants refused unhealthy-palatable foods or accepted to eat healthy-unpalatable foods, *PDI* is the Pupil Dilation Index, and *Task Order* is a factor accounting for the order in which the emotion regulation and dietary health challenge tasks were performed.

### Statistical analyses

All analyses were performed with the R^142^ and Matlab^143^ statistical software packages. For all Bayesian modelling analyses, we used the default, uninformative priors specified by the brms^144^ or BEST^74^ R-packages. The use of uninformative priors entails that our Bayesian analyses yield results very similar to those of conventional frequentist statistics. Results from all Bayesian analyses are reported as the mean of the posterior predictive distribution that indicates the most credible estimate, along with its standard deviation (SD) and the 95% Highest Density Interval that denotes the range in which 95% of the credible estimates fall. This is denoted as 95% Credible Interval (CI). The notation PP() indicates the posterior probability that the relation stated within the parentheses is true. Bayes Factors for the models of emotion regulation success were calculated with the R package “BayesFactor”. Bayes Factors are given for specific linear models comparing them to an intercept-only model. For the Bayesian equivalents of *t* tests (BEST) and correlations computed after Kruschke^74,145,146^, the corresponding functions to calculate Bayes Factors within these software packages were used.

## Supplementary Information

Supplementary Information.

## Data Availability

Data for the presented analyses  are available on OSF: https://osf.io/ub9gj/.

## Code availability

Code for the presented analyses is available on OSF: https://osf.io/ub9gj/.
