# Supplementary material for: Pupil dilation predicts individual self-regulation success across domains
Source: Sci Rep. 2021 Jul 12;11:14342. doi: 10.1038/s41598-021-93121-y (PMC8275757; doi:10.1038/s41598-021-93121-y)
Supplement: Supplementary file 1 — Supplementary Information. [file 41598_2021_93121_MOESM1_ESM.docx]

**Supplementary Information**

***for***

***Pupil dilation predicts individual self-regulation success across domains***

Silvia Maier ^1,2,3*+^, Marcus Grueschow ^1,2*+^

^1^ Zurich Center for Neuroeconomics, Department of Economics, University of Zurich

^2^ Neuroscience Center Zurich, University of Zurich, Swiss Federal Institute of Technology Zurich

^3^ Translational Neuromodeling Unit, Institute for Biomedical Engineering, University of Zurich and ETH Zurich

+ denotes equal contribution

* Correspondence should be addressed to:

silvia.maier@econ.uzh.ch

marcus.grueschow@econ.uzh.ch

***Supplementary Figures***

**Supplementary Figure 1.** Brightness matching of adaptation and target stimuli.


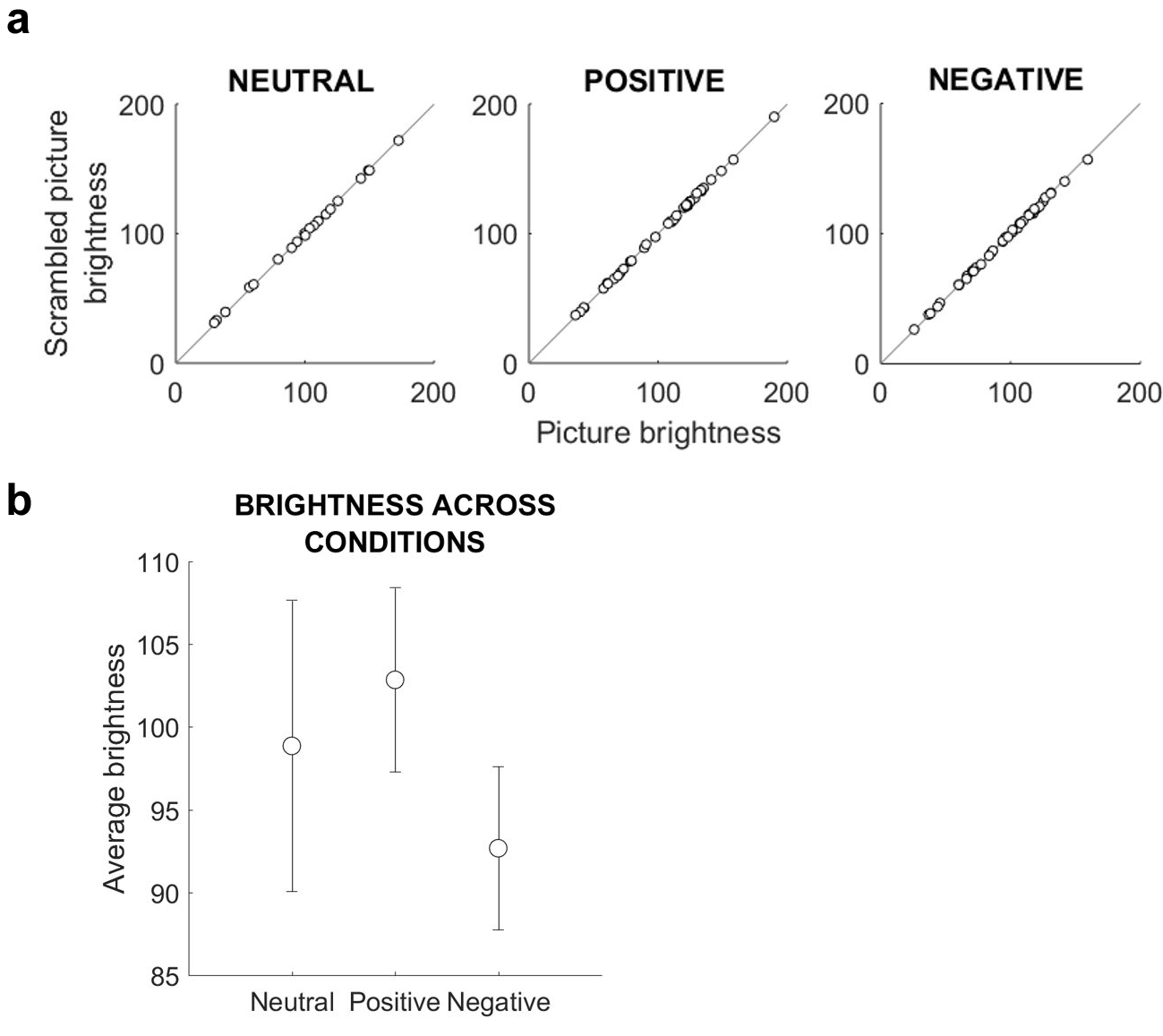


*Panel* ***a)*** *depicts the brightness-match of the adaptation and target stimuli for the neutral, positive and negative blocks. Each dot represents one stimulus. The 45-degree line is added for visual evaluation, indicating identity between the brightness of the scrambled adaptation stimuli and the target stimuli in which the content was visible.

Panel* ***b)*** *plots the mean brightness (dots) and the standard error of the mean (bars) for the stimuli in the neutral, positive and negative stimulus sets.*

**Supplementary Figure 2.** Results from the control analysis for regulation success prediction by blocks.

***
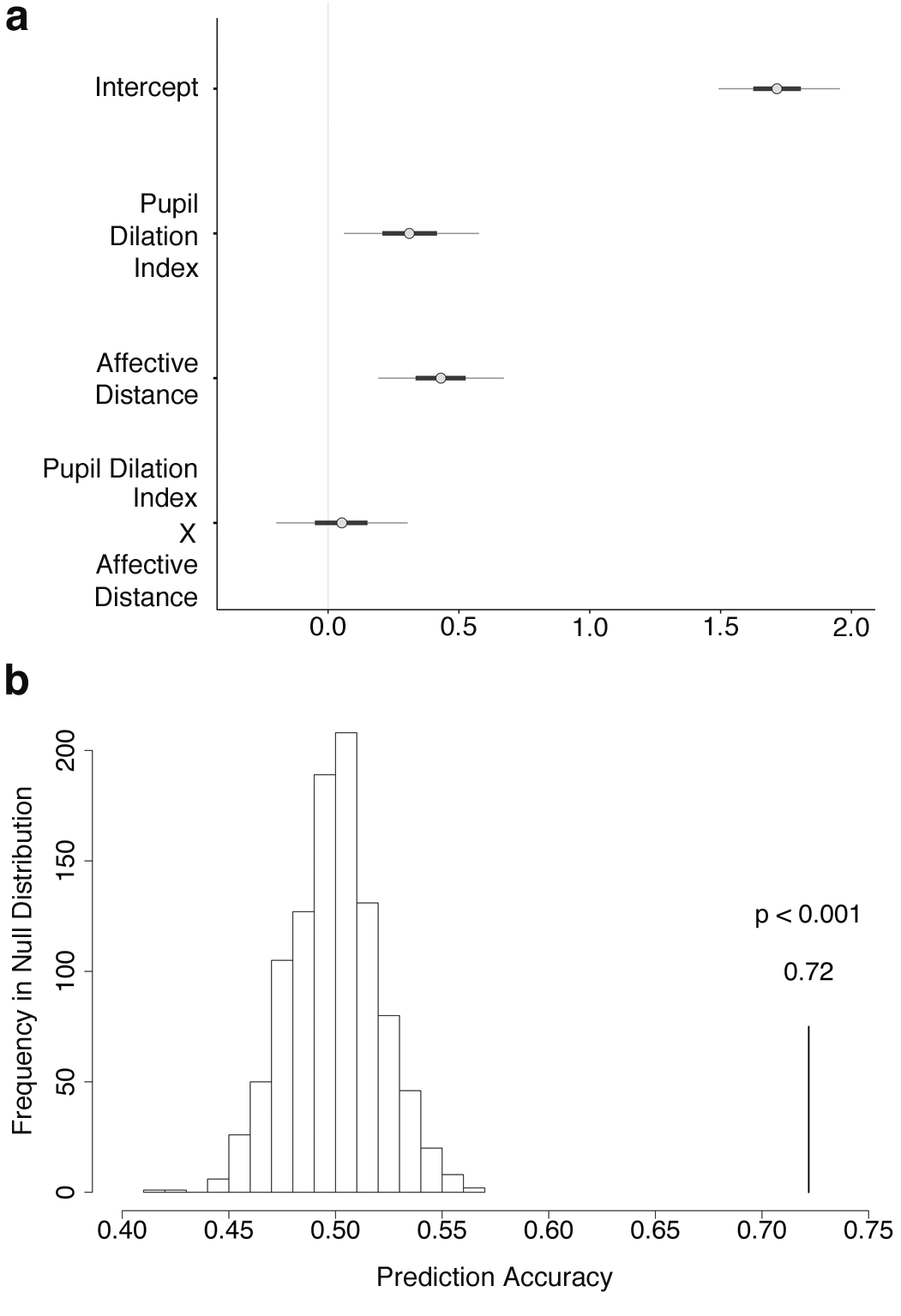
***

*Panel* ***a)*** *depicts the coefficients of the Bayesian linear regression fit of the model given in Eq. 3a for the control analysis using measures of reappraisal success and affective distance that were constructed from the view ratings given for the equivalent set of stimuli in the view block that had never been reappraised. As in the original model, we observed that regulation success increased both with greater Pupil Dilation Index and Affective Distance. The plot shows the mean beta estimates (grey dots) as well as the range of coefficients within the 90% Credible Interval that is represented by the light grey horizontal line (thick horizontal bars = 50% Credible Interval).

Panel* ***b)*** *shows the corresponding out-of-sample prediction. The results from above were cross-validated with a Leave-2-Participants-Out approach. Based on data from N-2 participants to which the above model was fit, we predicted which of the two left-out individuals regulated more successfully. The model predicted with 72% accuracy significantly above chance (p < 0.001).*

**Supplementary Figure 3.** Pupil diameter difference between Regulate and View trials (after preprocessing with a 200ms moving average window). **
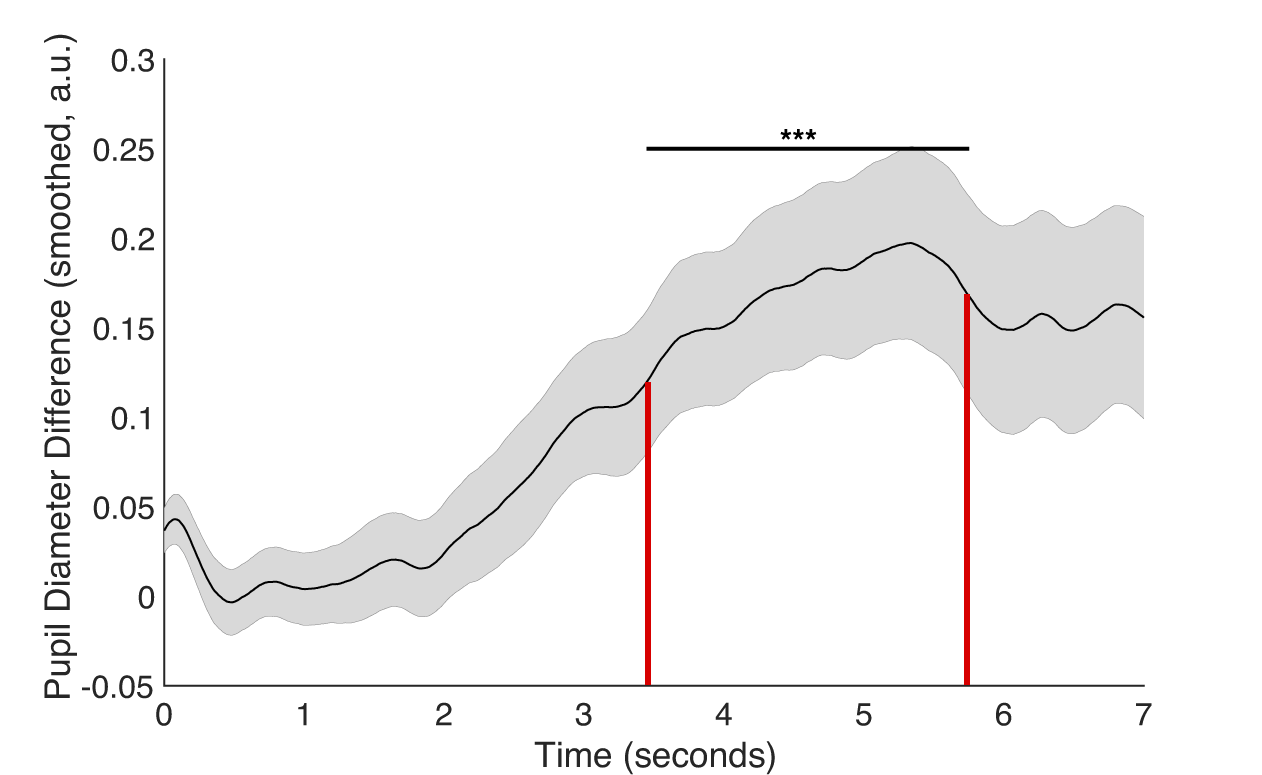
***Pupil Diameter Difference between Regulate and View Trials after preprocessing with a 200 ms moving average window. As in Figure 2b, we collapsed over positive and negative blocks in order to test for a valence-independent regulation signal across all participants. The mean z-scored pupil dilation for positive and negative view trials was subtracted from the mean z-scored dilation during positive and negative reappraise trials for each participant. A cluster-based permutation test indicated that between 3.4 and 5.7 seconds of the reappraisal/view period, the pupil dilation in reappraisal blocks was larger than during viewing blocks (p < 0.001; indicated by the black horizontal line and stars). For each participant, the mean of the Reappraisal – View difference in the pupil dilation signal during this period (marked by the red vertical lines in the plot) determined the pupil dilation index. The shaded areas indicate the standard error of the mean across participants. The black graph denotes the mean pupil dilation at each time point.*

***Supplementary Methods***

***Dataset.*** The pupil data we report here were recorded while acquiring an fMRI dataset that has first been reported in a companion paper by Maier and Hare ^1^. There, the authors tested an unrelated hypothesis using the fMRI and behavioural portions of the dataset. The present manuscript is the first report of the pupillometry data.

***Stimulus sets.*** Negative Set A: 1300, 2055.1, 2095, 2981, 3015, 3181, 3301, 3550, 6020, 6212, 6370, 6540, 6838, 9040, 9180, 9181, 9265, 9435, 9520, 9570; Negative Set B: 1525, 2352.2, 2683, 2800, 3051, 3250, 3530, 6312, 6415, 6570.1, 9140, 9250, 9252, 9253, 9300, 9430, 9561, 9571, 9635.1, 9800; Positive Set A: 1460, 1710, 1721, 1750, 1810, 1920, 2050, 2080, 2091, 2224, 2260, 2311, 2351, 2375.2, 2550, 5600, 5626, 5831, 5890, 8190; Positive Set B: 1440, 1463, 1720, 1731, 2058, 2071, 2150, 2170, 2303, 2345, 2620, 2655, 2660, 5390, 5594, 5628, 5830, 7580, 8461, 8497; Neutral Set: 2020, 2200, 2214, 2357, 2480, 2493, 2570, 2880, 2890, 7030, 7036, 7150, 7161, 7170, 7186, 7224, 7590, 7705, 7830, 8030***.***

***Reappraisal task instructions.*** To practice the reappraisal method, we provided standard written instructions that contained one example for positive and negative pictures before participants practiced modulating their emotional responses. Negative pictures for example contained scenes of humans suffering from wounds, war or crime scenes, or dead bodies of humans or animals. Positive scenes displayed animal or human babies, individuals playing or laughing, or nice views of landscapes. Participants were asked to modulate their interpretation of the pictures content such that negative feelings should become less negative, and positive feelings less positive. For example, one could think of the image as a scene or mock-up from a movie: Things are not as bad or good as they seem, but just staged. After receiving these standard instructions, participants trained with a computerized version of the task to familiarize themselves with the display sequence for one trial (adaptation stimulus – revealed stimulus – SAM rating screen), as it would be depicted in the actual experiment. First, participants reappraised one positive and one negative picture (order counterbalanced) at their own pace, and then two more images while picture presentation and emotion rating were presented with free timing and timed as in the actual experiment. Training was performed on pictures that were not presented in the actual experiment later. We assured participants felt competent to use the procedures before we started the recording as judged by mutual agreement between the participant and the experimenter.

***Control analysis for the reappraisal success measure.*** The “view” ratings of the reappraised images were collected after the scan session, i.e. after reappraising the content in the scanner. Previous work in the literature suggests there may be spillover effects from reappraisal in neural signals when the same stimuli are presented again ^2^, although these effects may be relatively transient and stronger when stimuli are reappraised multiple times ^3^. As a sanity check to exclude that any potential spillover effects from regulation would change our conclusions, we ran a control analysis following our method laid out before, but relying on the view ratings for the equivalent set of stimuli that were not reappraised but only viewed during the task.

Analogous to Equation 1, for the negative stimuli, we calculated for each participant according to Equation S2:

(Eq. S2) negative reappraisal success = mean reappraisal rating (reappraised negative set) - mean view rating (equivalent negative view set)

Analogous to Equation 2, for the positive stimuli, we calculated for each participant according to Equation S3:

(Eq. S3) positive reappraisal success = mean view rating (equivalent negative view set) - mean reappraisal rating (reappraised positive set)

To obtain the overall *regulation success score*, we then again took both success scores and calculated the mean across both positive and negative valence.

*Affective Distance* was also constructed using the view ratings from the view stimulus set for this control analysis. For each of the equivalent positive and negative stimulus sets we first averaged the mean difference of the view ratings from neutral, and then calculated the mean over the negative and positive sets.

With these measures, we then repeated the analysis described in Eq. 3a as well as the out-of-sample crossvalidation based on these regulation success measures.

***Stimulus construction for the pupil adaptation phase.*** Low-level feature matching was achieved via two image-processing steps. For any given trial, we first phase-scrambled the spatial frequency information of the target image (180°) using identical procedures as previously reported in Rieger, et al. ^4^. This approach renders the semantic content of the picture unrecognizable, while retaining the global contrast of the picture. The 180° phase-scrambled version of the target picture served as our adaptation stimulus. Such phase-scrambling methodology has previously been successfully employed to dissociate sensitivity to contrast and spatial frequency in human primary visual cortex ^4^. This step matched the contrast between the adaptation and target stimulus. This technique typically yields pictures that may appear darker than the original. Therefore the target image was brightness-adjusted (typically decreased by 10%) relative to the phase-scrambled version of the image using Matlab functions provided by Chris Rorden (https://www.mccauslandcenter.sc.edu/crnl/tools/bmp_contrast) and the Matlab Image Processing Toolbox. The result of the second image-processing step is thus equal global brightness distribution between the adaptation image and the target image, which contains semantic content to either be viewed or reappraised. Stimuli were presented at 126.5 cm viewing distance on a mean grey projection screen, with a stimulus size of 10.9 width x 8.1 cm height. Our procedure successfully matched the contrast and brightness for each pair of adaptation and target stimuli (Supplementary Figure 1), where plotting the brightness properties of the adaptation against the target stimuli results in almost perfect identity in brightness features.

***Control of visual stimulus properties.*** Across positive, negative and neutral emotion conditions, our stimulus sets may still differ in their low-level stimulus properties, as we applied the matching algorithm only within-trial. Indeed, we observed that on average, the pupil restricted more during the positive conditions than the negative (Figure 2A). In line with Henderson, et al. ^5^, this may be an effect of anticipating a threat in the negative condition, as stimuli were presented in blocks. The pupil also stayed on average more constricted throughout the positive view and regulation trials, which may be due to brightness differences of the stimuli. Although there was no difference at the significance level of p < 0.05, the average brightness for the positive stimuli tended to be higher than the average brightness of negative stimuli (Supplemental Fig. S1b; mean brightness positive = 102.85 cd/m^2^, mean brightness negative = 92.67 cd/m^2^, p = 0.18, 95% CI = [-4.80, 25.15] ). However, we control for any remaining differences with our design. In our main analysis of interest, we calculate a contrast between regulation and viewing that averages regulation and view signals over both positive and negative valence domains. Therefore, any brightness-induced differences in pupil size cancel out in this contrast, as both positive and negative stimuli appear on both sides of the subtraction, in the regulate or view condition. Hence the remaining differences are related to regulation, not the physical stimulus properties.

***Control analysis for saccades.*** To test for potential differences in pupil diameter due to saccades, we classified saccades as eye movements away from the center of the screen between 0.5 and 26.5 degrees visual angle. For each of these saccades, we logged the onset and counted the number in the negative and positive Regulate and View conditions for each participant. We then averaged the number of saccades during the Regulate and View conditions per participant and conducted Bayesian paired T-tests in order to test whether the number of saccades differed between the Regulate and View conditions. We also constructed a saccade index, analogously to our pupil dilation index for each participant, by subtracting the average number of saccades in the View condition from the average number of saccades in the Regulate condition. We then controlled for this saccade index in the regression model specified in eq. S4 in order to test whether the pupil dilation index continues to explain variance when accounting for the equivalent difference in saccades.

(Eq. S4) RS = β_0_ + β_1_ PDI + β_2_ Affective distance
+ Saccade Index + β_3_ PDI * Affective distance + *e*

***Testing a need for temporal filtering.*** Our presented analyses and results were all obtained using unfiltered data. We opted to not filter because we wanted to reduce degrees of freedom in potential analysis choices and most importantly, create a robust and simple analysis procedure to foster the ease of use and simplicity in translation to multiple and diverse settings for example in medical practice. To rule out that our results were influenced by physiologically unlikely spikes or rapid constrictions in the pupil data that are most likely resulting from noise, particularly when the sampling rate is relatively high as in this study, commonly temporal smoothing with a moving average is applied. Hence we tested in a supplementary analysis whether in addition to interpolation over blinks, filtering the pupil signal with a smoothing filter would change the results. We temporally smoothed the raw pupil signal with a 200 ms sliding time window using the Matlab-function movAvg.m with a type-parameter ‘simple’.

***Statistical packages.*** All Bayesian analyses were performed with the R ^6^, STAN ^7^ and JAGS ^8^ statistical software packages. Bayesian regressions were run using the brms package ^9^ that is an interface between R and STAN. All correlations ^10^ and t-tests were computed using Bayesian Markov Chain Monte Carlo (MCMC) sampling methods using R in combination with JAGS ^11^. The behavioural plots in Figure 1 were created using the yarrr package ^12^, the scatter plot in Figure 3C was created with ggplot ^13^. The package pracma ^14^ was used for data handling.

***Supplementary Results***

***Control analysis for the reappraisal success model.*** We quantified the emotion regulation success level mathematically based on the procedure by Wager, et al. ^15^. In order to exclude confounds due to potential spillover effects of reappraisal ^2^, we repeated the analysis described in Eq. 3a using measures for emotion reappraisal success and affective distance that were constructed block-wise, based on ratings given for the equivalent stimulus set used in the “view” condition. These measures were thus based on the mean view ratings from the equivalent negative and positive stimulus sets that had been equated with the reappraised stimulus sets for average arousal and valence. Like the reappraised images, these images in the view block had been viewed in the scanner for the first time. Using these measures, we found the same pattern of results as before (Supplementary Figure 2; Supplementary Table 2): the pupil dilation index explained a substantial portion of the reappraisal success (beta = 0.31 ± 0.16 SD, 95% Credible Interval (CI) = [0.01; 0.63]), above and beyond the effects of affective distance (beta = 0.43 ± 0.15, 95% CI = [0.13; 0.73]). The crossvalidation for this control analysis also corroborated our previous results (Supplementary Figure 2; prediction accuracy = 72%, p < 0.001). Hence our conclusions based on the more fine-grained, stimulus-wise constructed regulation success measure replicated.

***Control analysis for saccades.*** The mean number of saccades in the Regulate and View conditions did not differ (Mean Regulate = 517, Mean View = 510 saccades; PP (Difference in number of saccades between Regulate and View > 0) = 0.78; 95% HDI = [-12.17 26.59]; Bayes Factor (BF) = 0.11). The saccade index did not explain any variance in the emotion regulation success model (beta = -0.10 ± 0.14; 95% CI = [-0.38; 0.18]; BF for the model including the saccade index = 1.04; Supplementary Table 3), whereas the pupil dilation index continued to explain variance in emotion regulation success after accounting for potential saccade differences (beta = 0.32 ± 14; 95% CI = [0.04; 0.61]).

***PDI is independent of temporal filtering.*** We reconstructed the pupil dilation index using the temporally smoothed data and found virtually identical results for both the time window during which regulatory arousal was detected as well as for the individual values of the pupil dilation index per participant during this time frame. Starting and end points for the pupil dilation index were shifted by milliseconds: the onset of regulation in the unsmoothed data was detected at 3.419 seconds and in the smoothed data at 3.447 seconds (Supplementary Figure 3). The offset of the significant regulation period was detected at 5.608 seconds in the original data, and at 5.752 seconds in the temporally smoothed preprocessed dataset. We also tested the correlation between the individual pupil dilation index measures obtained without and with smoothing, and found an almost perfect correlation between the smoothed and unsmoothed data: Bayesian rho = 0.9921 (95% HDI = [0.991; 0.993]; PP(rho>0) > 0.9999; Bayes Factor > 10000). This additional analysis added yet another indicator for the robustness of our pupil dilation index and underlined the strength of our rigorously controlled design. These results indicate that future applicants of the methodology do not need to administer an additional smoothing pre-processing step.

***Supplementary Discussion***

***Development and rationale of the pupil dilation index.*** We determined the time window during which we identified regulation signals by calculating a cluster-based permutation t-test to separate the pupil signal related to regulating from the signal related to solely viewing emotional content without regulation demands. While this approach aims at incorporating all signals that relate to the regulation process, the within-participant subtraction ^16^ of “regulate” and “view” signals renders the remaining pupil dilation index robust and ensures that only regulation-relevant parts of the pupil dilation are evaluated in order to determine the timing. Because our design equated the stimulus sets between view and regulate conditions for their average valence and arousal, we thereby only evaluated those portions of the dilation response that go beyond the average dilation that was observed when emotional stimuli with similar properties were contemplated. Following the reasoning of Hess and Polt ^17^, who suggested that “total mental activity” could be captured as a combination of the amplitude and latency of the pupil dilation response, we then calculated the mean difference of the pupil dilation amplitude during the significant regulation time between the reappraisal and view condition to isolate the regulation-related components and determine the pupil dilation index we describe in this work (also see Kinner, et al. ^18^). This index thus captures all processes that are directly relevant to regulating.

***Prerequisites for constructing the pupil dilation index.*** To isolate the regulation-related signals in the pupil dilation time course, we followed a number of steps in our experimental design. These can be repeated with a broad range of stimuli for other cognitive experiments that go beyond the realm of emotion control. These prerequisites are: 1) Collecting norm ratings of the stimulus material in terms of arousal and valence beforehand, based on which 2) the experimental paradigm is constructed such that valence and arousal aspects are equated on average across the condition of interest and control condition, so that 3) any remaining portions of the pupil signature reflect the cognitive process(es) of interest that can be isolated by a subtraction contrast. 4) Adjusting the physical features of the stimuli across the sets for the control condition and the condition of interest in terms of brightness and contrast allows to interpret the subtraction contrast just in terms of the content features, and 5) the 1-second adaptation period with a phase-scrambled stimulus allows to interpret the pupil signal in continuous time from the start of the cognitive process of interest. Based on these prerequisites, 6) using a cluster-based permutation test facilitates tracking the cognitive process of interest in continuous time. Here, we employed a set of emotional stimuli with known valence and arousal norms, which have been successfully used in research through decades, but in principle, a large variety of stimulus material can undergo such norming to mimic our design.

***Potential reappraisal spillover.*** Previous work has shown that reappraisal may under certain conditions spill over on subsequent processing and rating of the stimuli. MacNamara, et al. ^2^ used standardized reappraisals that negatively framed unpleasant and neutral stimuli and found that these affected neural signals and ratings when presenting the same stimuli again 30 minutes later. However, the effects were more pronounced for neutral than negative stimuli. A study by Denny, et al. ^3^ presented negative stimuli in different scan sessions one day after first reappraisal and again a week later in a view or reappraise condition. In the reappraise condition, participants were to make their negative feelings less negative. Their results suggested that amygdala responses to negative stimuli were only attenuated in the long term after repeated reappraisal, but not when negative stimuli were only reappraised once. A study by Walter, et al. ^19^ found effects of reappraisal compared to viewing aversive stimuli that lasted up to 10 minutes after emotion regulation, and also suggested changed memory encoding when tested a year later ^20^. In summary, reappraisal effects might be relatively transient and stronger when stimuli are reappraised multiple times. In our experiment, participants re-rated the stimuli outside the scanner ca. 30-45 minutes after completing the last reappraisal session. To rule out potential concerns, we checked our results in a control analysis with measures constructed based on the “view condition” (i.e., an equivalent set of stimuli that had not been reappraised), and found that both the regression model as well as the out-of-sample cross-validation corroborated our previous results, ruling out reappraisal spillover as a main determinant of our results.

***Supplementary Tables***

**Supplementary Table 1.** Emotion valence ratings by condition.
*(Results in this table are reproduced from Maier and Hare ^1^)*

| **Condition** | **Mean  rating** | **Standard Deviation** |
| --- | --- | --- |
|  |  |  |
| Negative View | 2.69 | 0.54 |
| Negative Regulate | 4.25 | 0.81 |
| Neutral View | 5.26 | 0.41 |
| Positive Regulate | 5.21 | 0.90 |
| Positive View | 7.09 | 0.67 |

*Mean and standard deviation for the emotion ratings given in each condition. Valence ratings were recorded using a Self-Assessment Manikin Scale from 1 (very sad) to 9 (very happy) in steps of 1, with 5 being neutral.*

**Supplementary Table 2.** Control analysis: emotion regulation success score and affective distance calculated from block of not reappraised images.

|  | **Beta  Estimate** | **Standard Deviation** | **95% Credible Interval** |
| --- | --- | --- | --- |
| **(Intercept)** | **1.72** | **0.14** | **[1.44; 2.00]** |
| **Pupil Dilation Index** | **0.31** | **0.16** | **[0.01; 0.63]** |
| **Affective Distance** | **0.43** | **0.15** | **[0.13; 0.73]** |
| Pupil Dilation Index X Affective Distance | 0.05 | 0.16 | [-0.25; 0.36] |
| Bayes Factor (BF) | 1.09 |  |  |

*Results from the Bayesian Linear Regression specified in Eq. 3a. In this control analysis, we used the “view” condition blocks of stimuli that were not reappraised to construct the regulation success score and affective distance measures.*

*The emotion regulation success score was calculated as follows:*

*Analogous to Eq. 1, for the negative stimuli, we calculated for each participant their average rating after regulation in the negative condition and then subtracted their average rating after viewing the other, equivalent negative picture set that was presented in the scanner for mere viewing.*

*Analogous to Eq. 2, for the positive stimuli, we calculated for each participant the average rating for the other, equivalent positive picture set that was presented in the scanner for mere viewing and subtracted their average rating after regulation in the positive condition.*

*We then again took both success scores and calculated the mean regulation success score across both valences.*

*Affective Distance was constructed as well from the equivalent, non-reappraised view blocks. We first calculated for each of the positive and negative stimulus sets the mean difference of the view ratings from neutral, averaged it, and then computed the mean over the negative and positive sets.*

*The regulation success score was modelled by the mean-centred and standardized coefficients for Pupil Dilation Index (measured as mean difference in the pupil dilation curve for the Regulate > View contrast in the significant regulation time between 3.4 and 5.6 seconds) and Affective Distance. Pupil Dilation Index and Affective Distance were interacted, to test whether it is more or less effortful to regulate with smaller or greater affective distances. Model fits are given as the population level mean of the posterior distribution ± standard deviation (SD) and the 95% Credible Interval. The Bayes Factor (BF) is given for the comparison of this linear model compared to an intercept-only model.*

*As this table shows, compared to Table 1, the results and thus our conclusions remain qualitatively unchanged.*

**Supplementary Table 3.** Control analysis: emotion regulation success model controlling for potential saccade differences between the Regulate and View conditions.

|  | **Beta  Estimate** | **Standard Deviation** | **95% Credible Interval** |
| --- | --- | --- | --- |
| **(Intercept)** | **1.77** | **0.14** | **[1.49; 2.04]** |
| **Pupil Dilation Index** | **0.32** | **0.14** | **[0.04; 0.61]** |
| **Affective Distance** | **0.29** | **0.14** | **[0.01; 0.57]** |
| Saccade Index | -0.10 | 0.14 | [-0.38; 0.18] |
| Pupil Dilation Index X Affective Distance | 0.21 | 0.19 | [-0.16; 0.59] |
| BF | 1.04 |  |  |

*Results from the Bayesian Linear Regression specified in Eq. S4. We controlled in this model for a Saccade Index that quantifies the difference in the number of saccades between the Regulate and View condition as described below.*

*The regulation success score was modelled by the mean-centred and standardized coefficients for Pupil Dilation Index (measured as mean difference in the pupil dilation curve for the Regulate > View contrast in the significant regulation time between 3.4 and 5.6 seconds) and Affective Distance, controlling for the mean-centred and standardized coefficient for the Saccade Index (measured as the mean difference in the number of Saccades in the Regulate minus the View condition). Pupil Dilation Index and Affective Distance were interacted, to test whether it is more or less effortful to regulate with smaller or greater affective distances. Model fits are given as the population level mean of the posterior distribution ± standard deviation (SD) and the 95% Credible Interval. The Bayes Factor (BF) is given for the comparison of this linear model compared to an intercept-only model.*

*As this table shows, compared to Table 1, the results and thus our conclusions remain qualitatively unchanged by accounting for a potential difference in the number of saccades between the regulate and view condition.*

**References**

1 Maier, S. U. & Hare, T. A. BOLD activity during emotion reappraisal positively correlates with dietary self-control success. *Social Cognitive and Affective Neuroscience*, doi:10.1093/scan/nsaa097 (2020).

2 MacNamara, A., Ochsner, K. N. & Hajcak, G. Previously reappraised: the lasting effect of description type on picture-elicited electrocortical activity. *Social cognitive and affective neuroscience* **6**, 348-358 (2010).

3 Denny, B. T., Inhoff, M. C., Zerubavel, N., Davachi, L. & Ochsner, K. N. Getting over it: Long-lasting effects of emotion regulation on amygdala response. *Psychological Science* **26**, 1377-1388 (2015).

4 Rieger, J. W. *et al.* BOLD responses in human V1 to local structure in natural scenes: Implications for theories of visual coding. *J Vis* **13**, 19, doi:10.1167/13.2.19 (2013).

5 Henderson, R. R., Bradley, M. M. & Lang, P. J. Modulation of the initial light reflex during affective picture viewing. *Psychophysiology* **51**, 815-818, doi:10.1111/psyp.12236 (2014).

6 R Core Team. *R: A Language and Environment for Statistical Computing*. (2013).

7 Carpenter, B. *et al.* Stan: A probabilistic programming language. *Journal of Statistical Software* **20**, 1-37 (2016).

8 Plummer, M. in *Proceedings of the 3rd international workshop on distributed statistical computing.* (eds Kurt Hornik, Friedrich Leisch, & Achim Zeileis).

9 Bürkner, P.-C. brms: An R Package for Bayesian Multilevel Models Using Stan. *Journal of Statistical Software* **80**, 1-28, doi:10.18637/jss.v080.i01 (2017).

10 Kruschke, J. K. *Doing Bayesian data analysis: A tutorial with R, JAGS, and Stan*. Second edn, (Academic Press / Elsevier, 2015).

11 Kruschke, J. K. Bayesian estimation supersedes the t test. *J. Exp. Psychol. Gen.* **142**, 573-603, doi:10.1037/a0029146 (2013).

12 yarrr: A Companion to the e-Book "YaRrr!: The Pirate's Guide to R" v. R package version 0.1.5 (2017).

13 The tidyverse v. 3.2.0 (2017).

14 pracma: Practical Numerical Math Functions v. R package version 2.1.4 (2018).

15 Wager, T. D., Davidson, M. L., Hughes, B. L., Lindquist, M. A. & Ochsner, K. N. Prefrontal-subcortical pathways mediating successful emotion regulation. *Neuron* **59**, 1037-1050, doi:10.1016/j.neuron.2008.09.006 (2008).

16 van der Wel, P. & van Steenbergen, H. Pupil dilation as an index of effort in cognitive control tasks: A review. *Psychon Bull Rev* **25**, 2005-2015, doi:10.3758/s13423-018-1432-y (2018).

17 Hess, E. H. & Polt, J. M. Pupil Size in Relation to Mental Activity during Simple Problem-Solving. *Science* **143**, 1190-1192, doi:10.1126/science.143.3611.1190 (1964).

18 Kinner, V. L. *et al.* What our eyes tell us about feelings: Tracking pupillary responses during emotion regulation processes. *Psychophysiology* **54**, 508-518, doi:10.1111/psyp.12816 (2017).

19 Walter, H. *et al.* The temporal dynamics of voluntary emotion regulation. *PLoS one* **4**, e6726 (2009).

20 Erk, S., Von Kalckreuth, A. & Walter, H. Neural long-term effects of emotion regulation on episodic memory processes. *Neuropsychologia* **48**, 989-996 (2010).
